# Dedifferentiated Osteosarcoma of the Wrist With BCOR Sarcoma Component in a 22‐Year‐Old Patient: A Case Report

**DOI:** 10.1155/cro/9962833

**Published:** 2026-06-25

**Authors:** Alba Hernandez-Guzman, Binyam Fentaw, Kassaye Firde, Shuanzeng Wei, Daniela Proca

**Affiliations:** ^1^ Department of Pathology, Temple University Hospital, Philadelphia, Pennsylvania, USA, temple.edu; ^2^ Department of Pathology, Fox-Chase Cancer Center, Philadelphia, Pennsylvania, USA

**Keywords:** BCOR, cytogenomics, molecular pathology, sarcoma, osteosarcoma

## Abstract

We report a unique case of a 22‐year‐old male with a progressively enlarging mass on the right thumb. Initial biopsies suggested a low‐grade osteogenic tumor. Following partial amputation, gross examination revealed a well‐circumscribed 3.7cm mass involving the metacarpal bone with soft tissue invasion. Histologically, the tumor displayed features of low‐grade central osteosarcoma with areas transitioning to a round blue cell morphology resembling Ewing sarcoma. Comprehensive molecular testing—including next‐generation sequencing (NGS) and cytogenomic microarray analysis (CMA)—identified amplifications of MDM2 and CDK4, hallmark alterations of low‐grade osteosarcoma. Notably, a BCOR internal tandem duplication (ITD) was also detected. The final diagnosis was central low‐grade osteosarcoma with dedifferentiation to BCOR‐altered sarcoma. To our knowledge, this is the first reported case of osteosarcoma with BCOR sarcoma dedifferentiation occurring in the wrist of a young adult. BCOR ITD sarcomas have previously been described exclusively in nonappendicular soft tissues of infants and young children, without known association with osteosarcoma. This case highlights the diagnostic value of integrating NGS (DNA and RNA) and CMA in sarcomas with mixed or unusual histology. Such tools are essential for precise classification, prognostication, and consideration of targeted therapies in rare and histologically complex bone tumors.

## 1. Introduction

Dedifferentiated osteosarcoma (OS) is an uncommon and aggressive malignancy characterized by the coexistence of a high‐grade sarcomatous component originating from a pre‐existing low‐grade OS. This result in a more rigorous clinical regimen with a poorer prognosis compared with conventional OS. This highlights the importance of timely diagnosis and intervention [1]. The high‐grade component can morphologically display spindle cell or pleomorphic features, overlapping with other sarcoma subtypes, making the diagnostic process more difficult [1–6].

BCOR‐altered sarcomas and related tumors represent a molecularly defined group characterized by recurrent genetic events involving the BCOR gene, including most commonly BCOR::CCNB3, as well as BCOR internal tandem duplication and other BCOR rearrangements such as ZC3H7B::BCOR [6–10]. These alterations define a spectrum of neoplasms that share overlapping transcriptional programs and immunophenotypic features rather than a single uniform entity. In addition, a molecularly related subgroup characterized by YWHAE::NUTM2 fusions demonstrates similar morphologic and transcriptional features, including BCOR overexpression, despite lacking direct BCOR genetic alterations [11–13]. Histologically, these tumors most often exhibit a primitive round cell to spindle cell morphology, with variability ranging from highly cellular round cell patterns (typical of BCOR::CCNB3 sarcomas) to more spindle cell and fibromyxoid appearances (seen in BCOR‐rearranged uterine sarcomas and BCOR ITD tumors) [2, 4, 6]. Immunohistochemically, strong nuclear BCOR expression is a common and diagnostically useful feature across this spectrum, reflecting the central role of BCOR—a transcriptional corepressor—in oncogenesis [8–10].

Treatment strategies for these tumors typically involve surgical resection with wide margins, and adjunct systemic chemotherapy [4–8, 14, 15]. Large collaborative studies have established BCOR‐rearranged sarcomas as a molecularly distinct, but histologically heterogeneous group of ultra‐rare round cell neoplasms with overlapping morphologic features and variable clinical behavior, underscoring both their diagnostic complexity and the lack of standardized therapeutic approaches [14, 15]. Although a standard chemotherapy regimen has yet to be established for BCOR‐associated sarcomas, existing Ewing sarcoma protocols have shown benefit under specific conditions [5–8] [14, 15]. The prognosis is influenced by the degree of dedifferentiation, specific genetic alterations, and potential for complete surgical resection.[1],[4–10] [14–18],

## 2. Case Presentation

**Figure 1:**
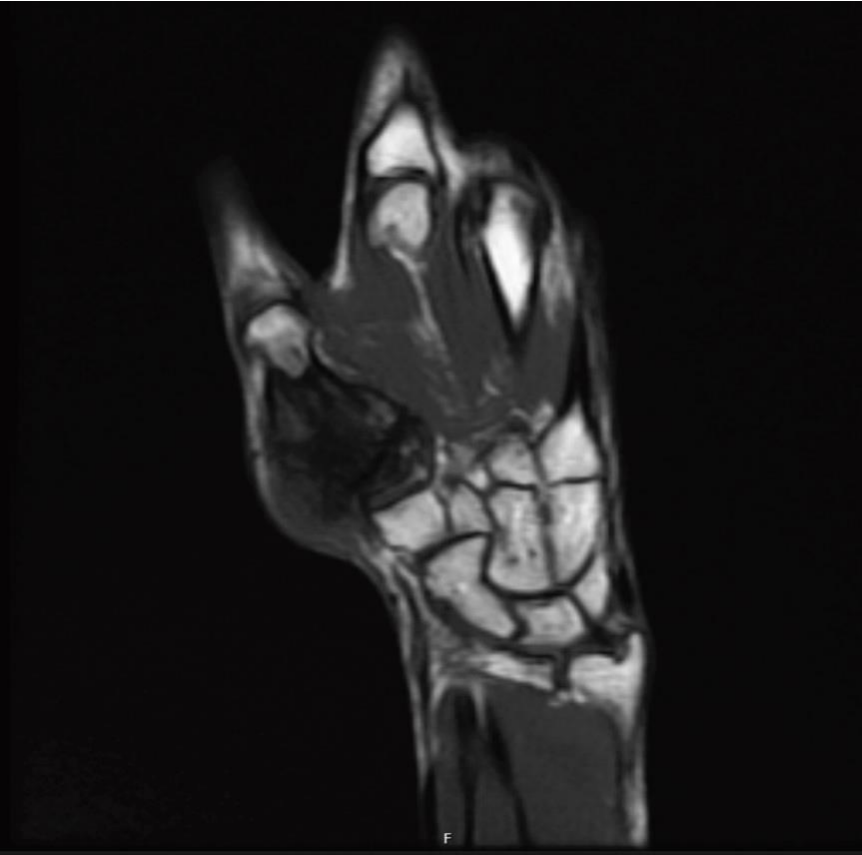
MRI of right hand showing a destructive mass involving the first metacarpal bone.

This is the case of a 22‐year‐old male who presented with a progressively enlarging mass on the right thumb at a previous fracture site—the fracture occurred 5 years before the current admission and the mass was initially noticed within the first couple years; the mass was noted to enlarge rapidly over 6–8 months prior to current admission. An MRI of the hand (Figure 1) showed a destructive mass involving the first metacarpal bone—the mass had mixed signal intensity, with areas of T2 hyperintense signal and internal low signal intensity. Two needle core biopsies performed had pathology suggesting a low‐grade osteogenic tumor. The two biopsies showed crushed spindled and round cells with focal bone formation and mild atypia, as well as scattered multinucleated giant cells; the neoplastic cells were positive with CD99, vimentin, and focally Sat‐B2; they were negative with S100, EMA, HHF35, AE1/AE3, CD68, synaptophysin, and chromogranin. Next‐generation sequencing (NGS) and cytogenomic microarray analysis (CMA) were attempted on both biopsies, but the material was inadequate for testing, likely secondary to decalcification. Decalcification, even when performed using slow chelating agents such as EDTA, can degrade nucleic acids and disrupt nuclear integrity, thereby reducing the sensitivity and reliability of NGS, FISH, and CMA.

PET and MRI showed no evidence of metastases, and the patient underwent partial amputation of the right thumb, and iliac crest bone grafting was performed. Gross examination of the specimen showed a well circumscribed, 3.7 cm mass, involving the metacarpal bone, focally eroding through the cortex and periosteum, and infiltrating into adjacent soft tissue.

**Figure 2:**
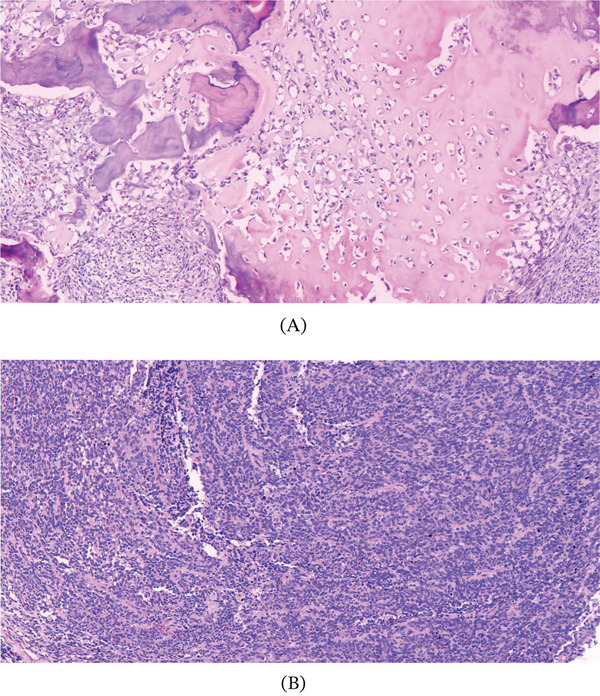
H&E, original magnification ×100 A (upper panel) and ×200 B (lower panel). Microscopic appearance of the two tumor components. (A) Osteosarcoma composed of spindled and osteoblast‐like tumor cells with mild nuclear atypia and increased cellularity, accompanied by lace‐like osteoid deposition that is focally hypercellular with atypical nuclei. (B) Blue round cell component with primitive “Ewing‐like” morphology, composed of sheets of round‐oval blue cells with high nuclear‐to‐cytoplasmic ratio, finely dispersed chromatin, and inconspicuous nucleoli, corresponding to the BCOR‐ITD–associated sarcomatous component.

Microscopic evaluation demonstrated two distinct components: a central OS and a peripheral blue round cell/Ewing sarcoma‐like/ES‐like (Figure 2). The intraosseous component consisted of OS with lace‐like osteoid production and osteoblast‐like tumor cells, showing nuclear expression of MDM2 and CDK4 by immunohistochemistry. The peripheral part of the tumor, extending into soft tissue, displayed a primitive blue cell morphology/ES‐like. Notably, CD99 and MDM2/CDK4 showed focal positivity across multiple tested blocks, involving both the OS and ES‐like components. MDM2 FISH performed on the intraosseous component was unsuccessful, likely due to decalcification‐related DNA degradation.

Molecular studies, DNA and RNA NGS, as well as CMA, were performed on non‐decalcified peripheral tumor tissue and demonstrated an in‐frame BCOR internal tandem duplication, supporting classification as a BCOR‐altered sarcoma. CMA and NGS RNA revealed a complex copy‐number profile with multiple segmental gains and losses, consistent with secondary genomic evolution. Additional DNA variants (TNFRSF14, GEN1, CUX1, FLCN, and BCR) were of uncertain significance and do not supersede BCOR‐ITD as the likely driver alteration (Tables 1 and 2).

**Table 1:** Cytogenomic microarray findings (CMA).

Chromosomal region	Coordinates (hg19)	Copy number	Type of alteration	Notes
1p36.33–p34.3	0.75–35.8 Mb	x1	Loss	Distal 1p loss
1p34.3	35.8–38.0 Mb	x3	Gain	Focal gain
1p34.3–p32.1	39.4–59.1 Mb	x3	Gain	Segmental gain
1p32.1–p21.3	61.2–173.6 Mb	x4	High‐level gain	Broad amplification
1q25.1–q32.1	173.6–200.1 Mb	x3	Gain	Segmental gain
1q32.1–q44	203.8–249.2 Mb	x4	High‐level gain	Distal amplification
2p22.2–q37.3	37.8–242.9 Mb	x3	Gain	Near whole chromosome gain
5p	—	x3	Gain	Whole arm gain
5q31.2–q35.3	139.1–180.7 Mb	x3	Gain	Includes oncogenic loci
6q16.1–q21	95.2–110.8 Mb	x3	Gain	Segmental gain
7p22.2–q36.3	2.8–159.1 Mb	x3	Gain	Near whole chromosome gain
10p15.3–p14	0.1–11.7 Mb	x1	Loss	Focal deletion
10q24.2–q26.3	101.2–134.7 Mb	x1	Loss	Distal loss
17q24.3–q25.3	67.5–81.1 Mb	x3	Gain	Segmental gain

*Note:* Overall interpretation: Abnormal karyotype with multiple segmental gains and losses, present in ~40%–60% of cells. The tumor demonstrates a complex copy‐number profile with multiple chromosomal gains and losses, consistent with a genetically unstable malignant clone with subclonal heterogeneity. No single canonical osteosarcoma‐defining amplification (e.g., isolated MDM2/CDK4‐driven pattern) is identified, supporting a genomically complex sarcoma rather than a classic low‐grade osteosarcoma profile.

**Table 2:** DNA variants (NGS Panel).

Gene	Protein change	cDNA change	VAF (%)	Variant type	Interpretation
TNFRSF14	p.Gly168Glu	c.503G>A	24.1	Missense	Likely subclonal, uncertain significance
GEN1	p.Ser509Leu	c.1526C>T	48.3	Missense	Near heterozygous, unclear role
CUX1	p.Ile543Val	c.1627A>G	31.7	Missense	Possible tumor‐associated, low specificity
FLCN	p.Pro311Ser	c.931C>T	29.6	Missense	Likely incidental/VUS
BCR	p.Phe1267fs	c.3800_3803del	49.1	Frameshift	Disruptive but not lineage‐defining

*Note:* Overall interpretation: The detected sequence variants are of uncertain or secondary significance and do not define tumor lineage. None represent established driver alterations for osteosarcoma or BCOR‐associated sarcoma, and collectively they are best interpreted as passenger or subclonal events in the context of a genomically complex neoplasm.

Taken together, the morphologic and molecular findings support central OS with dedifferentiation into a BCOR‐ITD–associated sarcoma. Although separate molecular analysis of each component would further substantiate this relationship, this was not feasible, as the intraosseous portion required decalcification and proved repeatedly inadequate for testing despite multiple attempts.

After uneventful surgery, the patient received adjuvant chemotherapy (vincristine, doxorubicin, and cyclophosphamide alternating with ifosfamide and etoposide; 11 cycles) and remains well, with no evidence of recurrence or metastasis at 20 months follow‐up.

## 3. Discussion

A dedifferentiated OS with a BCOR sarcoma component occurring in the wrist of a 22‐year‐old is exceptionally rare, both in terms of tumor biology and clinical presentation [5–9]. Dedifferentiated OS itself is an uncommon and aggressive variant of OS, typically characterized by a low‐grade conventional OS component alongside a high‐grade sarcomatous component [1]. Anatomically, OS most commonly arises in the metaphyseal regions of long bones, particularly around the knee, such as the distal femur or proximal tibia, or occasionally in the proximal humerus. The distal radius and wrist represent an atypical location for primary OS, accounting for less than 1% of cases. Thus, the combination of dedifferentiated OS and a wrist location already sets this case apart as an anomaly in the common spectrum of presentations.

What makes this case even more unusual is the presence of a BCOR‐mutated sarcomatous component within the dedifferentiated portion of the tumor. BCOR (BCL6 corepressor) gene alterations are associated with a distinct group of high‐grade undifferentiated sarcomas and other rare tumors such as clear cell sarcoma of the kidney and primitive myxoid mesenchymal tumor of infancy [5–8]. BCOR‐altered sarcomas tend to present in infants and children [4–9], whereas dedifferentiated OS usually affects young adults [1].

A focused review of the literature does not identify any well‐documented cases of OS dedifferentiating into a BCOR‐altered sarcoma. Instead, available studies consistently support a different phenomenon—namely, that BCOR‐altered sarcomas of bone, including those harboring BCOR::CCNB3 fusions or BCOR internal tandem duplications, may closely mimic OS, particularly small cell OS, at both histologic and radiologic levels.[3–7] [15–19], Recent studies emphasize that BCOR‐altered sarcomas belong to a group of genetically defined round cell neoplasms that frequently overlap morphologically with other entities, including Ewing sarcoma and small cell OS. Li et al. [16] demonstrated that BCOR‐rearranged sarcomas are commonly misclassified on histologic grounds alone and highlighted the critical role of molecular testing in establishing the correct diagnosis [16]. Similarly, Yoshida′s comprehensive review underscores that Ewing and Ewing‐like sarcomas, including those with BCOR alterations, represent a molecularly defined spectrum in which morphology may range from round to spindle cell patterns and may occasionally include divergent features such as focal matrix production [17]. In a recent clinicopathologic and molecular analysis, cases initially diagnosed as small cell OS were reclassified following molecular testing, including tumors with BCOR alterations, highlighting the diagnostic overlap rather than true lineage progression or dedifferentiation [18]. Furthermore, larger series of BCOR‐altered sarcomas emphasize their frequent presentation in bone and their potential to exhibit focal matrix production, contributing to misclassification as OS [17–19]. In contrast, the established model of dedifferentiated OS remains characterized by progression from low‐grade OS with MDM2/CDK4 amplification to a high‐grade sarcomatous component, without evidence of BCOR‐driven transformation [20]. Together, all these studies support the concept that BCOR‐altered sarcomas are important histologic mimics of other primary bone tumors, reinforcing the need for molecular characterization in diagnostically challenging cases.

To our knowledge, this represents the first case to propose a low‐grade OS undergoing dedifferentiation into a BCOR ITD‐associated sarcoma. In this context, our case is unique. Histologically, it demonstrates two distinct but spatially related components: a central region showing osteoid production with osteoblast‐like neoplastic cells expressing SATB2, CDK4, and MDM2, consistent with low‐grade OS, and a peripheral component with a primitive round cell (“Ewing‐like”) morphology harboring a BCOR internal tandem duplication on molecular testing (Figure 2, Tables 1 and 2). Although BCOR‐altered sarcomas are well recognized to mimic small cell OS, the OS component in our case is clearly not of the small cell type and instead represents a histologically distinct osteogenic neoplasm coexisting with a BCOR‐ITD–associated sarcoma with ES‐like classic histology. This biphasic pattern, supported by morphologic and molecular findings, highlights the uniqueness of this case.

Given the diagnostic complexity of this case, management was guided by a multidisciplinary team (MDT) discussion involving pathology, orthopedic oncology, radiology, and medical oncology. The integration of histologic, immunophenotypic, radiologic, and molecular findings was essential in reaching the final diagnosis and formulating the treatment plan. In particular, recognition of the BCOR‐altered component influenced therapeutic decision‐making, supporting the use of systemic chemotherapy in addition to surgical resection. In addition to proposing a first OS‐ BCOR ITD sarcoma association, this case highlights the critical role of a coordinated MDT approach in the evaluation and management of diagnostically challenging bone tumors.

Finally, our report is intended to increase awareness to the complexity in managing OS‐BCOR ITD associated sarcomas if we accept the possibility that they can coexist. These tumors present both diagnostic and therapeutic challenges, as they combine features of distinct biologic entities with distinct clinical behaviors and treatment paradigms. As such, cases with mixed osteogenic and BCOR‐altered features highlight the need for careful integration of histologic, immunophenotypic, and molecular data to guide management and attempt to standardize treatment for these rare and complex neoplasms.

## Funding

No funding was received for this manuscript.

## Ethics Statement

This is a case report; IRB approval was not necessary.

## Consent

Consent to “take photographs for the purpose of medical study or research, and the reproduction or publication of these photographs in any manner, providing the patient′s identity is not revealed” was obtained in writing and is available in the patient′s medical records. NOTE: The IRB does not require consent forms for Pathology Case Reports.

## Conflicts of Interest

The authors declare no conflicts of interest.

## Data Availability

Additional data is not largely available due to HIPAA; all available data is presented in this report.
